# [Retracted] MicroRNA‑15b promotes proliferation and invasion of non‑small cell lung carcinoma cells by directly targeting TIMP2

**DOI:** 10.3892/or.2023.8611

**Published:** 2023-08-07

**Authors:** Haowen Wang, Yu Zhan, Jingjing Jin, Chunhong Zhang, Wenfeng Li

Oncol Rep 37: 3305–3312, 2017; DOI: 10.3892/or.2017.5604

Following the publication of this paper, it was drawn to the Editor's attention by a concerned reader that certain of the cell migration and invasion images shown in Figs. 3B and D and 6C were strikingly similar to data that had already appeared in an article written by different authors at different research institutes [Liu C, Guan H, Wang Y, Chen M, Xu B, Zhang L, Lu K, Tao T and Zhang X: miR-195 inhibits EMT by targeting FGF2 in prostate cancer cells. PLoS One 9: e0144073, 2015].

Owing to the fact that the contentious data in the above article had already been published prior to its submission to *Oncology Reports*, the Editor has decided that this paper should be retracted from the Journal. The authors were asked for an explanation to account for these concerns, but the Editorial Office did not receive a reply. The Editor apologizes to the readership for any inconvenience caused.

